# Supplementary material for: Resolving the full spectrum of human genome variation using Linked-Reads
Source: Genome Res. 2019 Apr;29(4):635–45. doi: 10.1101/gr.234443.118 (PMC6442396; doi:10.1101/gr.234443.118)
Supplement: Supplemental Material [file supp_gr.234443.118_Supplemental_longranger-2.2.zip › longranger-2.2.tar.bz2!/lr2.2-public/lib/stan/cmdstan/stan/doc/api/html/structdata__only__expression.html]

Stan: data\_only\_expression Struct Reference


|  |  |
| --- | --- |
|  | Stan  2.5.0  probability, sampling & optimization |


- Main Page
- Related Pages
- Namespaces
- Classes
- Files

- Class List
- Class Index
- Class Hierarchy
- Class Members

All Classes Namespaces Files Functions Variables Typedefs Enumerations Enumerator Friends Macros Pages

Public Member Functions |
Public Attributes |
List of all members

data\_only\_expression Struct Reference

`#include <var_decls_grammar_def.hpp>`

Inheritance diagram for data\_only\_expression:

|  |  |
| --- | --- |
| Public Member Functions | |
|  | data\_only\_expression (std::stringstream &error\_msgs, variable\_map &var\_map) |
|  | |
| bool | operator() (const nil &) const |
|  | |
| bool | operator() (const int\_literal &) const |
|  | |
| bool | operator() (const double\_literal &) const |
|  | |
| bool | operator() (const array\_literal &x) const |
|  | |
| bool | operator() (const variable &x) const |
|  | |
| bool | operator() (const integrate\_ode &x) const |
|  | |
| bool | operator() (const fun &x) const |
|  | |
| bool | operator() (const index\_op &x) const |
|  | |
| bool | operator() (const binary\_op &x) const |
|  | |
| bool | operator() (const unary\_op &x) const |
|  | |

|  |  |
| --- | --- |
| Public Attributes | |
| std::stringstream & | error\_msgs\_ |
|  | |
| variable\_map & | var\_map\_ |
|  | |

## Detailed Description

Definition at line 172 of file var\_decls\_grammar\_def.hpp.

## Constructor & Destructor Documentation

|  |  |  |  |
| --- | --- | --- | --- |
| data\_only\_expression::data\_only\_expression | ( | std::stringstream & | *error\_msgs*, |
|  |  | variable\_map & | *var\_map* |
|  | ) |  |  |

inline

Definition at line 175 of file var\_decls\_grammar\_def.hpp.

## Member Function Documentation

|  |  |  |  |  |  |
| --- | --- | --- | --- | --- | --- |
| bool data\_only\_expression::operator() | ( | const nil & |  | ) | const |

inline

Definition at line 180 of file var\_decls\_grammar\_def.hpp.

|  |  |  |  |  |  |
| --- | --- | --- | --- | --- | --- |
| bool data\_only\_expression::operator() | ( | const int\_literal & |  | ) | const |

inline

Definition at line 183 of file var\_decls\_grammar\_def.hpp.

|  |  |  |  |  |  |
| --- | --- | --- | --- | --- | --- |
| bool data\_only\_expression::operator() | ( | const double\_literal & |  | ) | const |

inline

Definition at line 186 of file var\_decls\_grammar\_def.hpp.

|  |  |  |  |  |  |
| --- | --- | --- | --- | --- | --- |
| bool data\_only\_expression::operator() | ( | const array\_literal & | *x* | ) | const |

inline

Definition at line 189 of file var\_decls\_grammar\_def.hpp.

|  |  |  |  |  |  |
| --- | --- | --- | --- | --- | --- |
| bool data\_only\_expression::operator() | ( | const variable & | *x* | ) | const |

inline

Definition at line 195 of file var\_decls\_grammar\_def.hpp.

|  |  |  |  |  |  |
| --- | --- | --- | --- | --- | --- |
| bool data\_only\_expression::operator() | ( | const integrate\_ode & | *x* | ) | const |

inline

Definition at line 210 of file var\_decls\_grammar\_def.hpp.

|  |  |  |  |  |  |
| --- | --- | --- | --- | --- | --- |
| bool data\_only\_expression::operator() | ( | const fun & | *x* | ) | const |

inline

Definition at line 214 of file var\_decls\_grammar\_def.hpp.

|  |  |  |  |  |  |
| --- | --- | --- | --- | --- | --- |
| bool data\_only\_expression::operator() | ( | const index\_op & | *x* | ) | const |

inline

Definition at line 220 of file var\_decls\_grammar\_def.hpp.

|  |  |  |  |  |  |
| --- | --- | --- | --- | --- | --- |
| bool data\_only\_expression::operator() | ( | const binary\_op & | *x* | ) | const |

inline

Definition at line 229 of file var\_decls\_grammar\_def.hpp.

|  |  |  |  |  |  |
| --- | --- | --- | --- | --- | --- |
| bool data\_only\_expression::operator() | ( | const unary\_op & | *x* | ) | const |

inline

Definition at line 233 of file var\_decls\_grammar\_def.hpp.

## Member Data Documentation

std::stringstream& data\_only\_expression::error\_msgs\_

Definition at line 173 of file var\_decls\_grammar\_def.hpp.

variable\_map& data\_only\_expression::var\_map\_

Definition at line 174 of file var\_decls\_grammar\_def.hpp.

---

The documentation for this struct was generated from the following file:

- src/stan/gm/grammars/var\_decls\_grammar\_def.hpp


---

[ Stan Home Page ]

*© 2011–2014,
Stan Development Team.*
